# Laparoscopic treatment of a recurrent biliary stone forming around a Hem-o-lok clip in a patient with previous gastrectomies: Case report

**DOI:** 10.1097/MD.0000000000027213

**Published:** 2021-09-24

**Authors:** Chao Jiang, Xueyan Liu, Shuxuan Li, Guangzhen Wu, Guangyi Wang, Meng Wang

**Affiliations:** a Department of Hepatobiliary Pancreatic Surgery, the First Hospital of Jilin University, Changchun, Jilin Province, China; b Cardiovascular Department, China-Japan Union Hospital of Jilin University, Changchun, Jilin Province, China.

**Keywords:** case report, clip migration, gastrectomy, laparoscopic common bile duct exploration, primary closure

## Abstract

**Rationale:**

A history of gastrectomy is associated with an increased incidence of gallstones requiring surgery. Endoscopic retrograde cholangiopancreatography is challenging for patients who undergo total or Billroth II gastrectomy. Laparoscopic common bile duct exploration (LCBDE) has been attempted in such cases. Herein, we report a case of choledocholithiasis in which a stone formed around a migrated Hem-o-lok clip.

**Patient concerns:**

A 67-year-old man was admitted to the hospital for acute right upper abdominal pain. He had a history of 2 open gastric cancer surgeries in the previous seven years and had undergone LCBDE 12 months prior to this admission. Postoperative examination revealed recurrence of bile duct stones.

**Interventions:**

The patient underwent repeat LCBDE plus primary closure with an evaluation of abdominal adhesion. A stone had formed around a Hem-o-lok clip in the common bile duct was removed.

**Outcomes:**

The patient had an uneventful recovery with no stone recurrence or movement of the remaining Hem-o-lok clips after a 1-year follow-up.

**Lessons:**

LCBDE with primary closure should be carefully considered in patients with certain gallstone diseases after complicated upper abdominal surgery.

Postoperative clip migration is a rare complication; hence care must be taken in placing the clip appropriately to ensure that it is not too close to the common bile duct.

## 1 Introduction

Patients with a history of gastrectomy have an increased incidence of gallstones and gallbladder morbidity requiring surgery.^[1–3]^ However, as one of the most commonly selected therapies for gallbladder disease, endoscopic retrograde cholangiopancreatography (ERCP) with stone extraction combined with laparoscopic cholecystectomy (LC) is challenging to perform because of the requirement for the reconstruction of the digestive tract and the associated extensive adhesions. This treatment not only demands high technology^[4]^ but also requires a 2-stage approach and can lead to both ERCP- and laparoscopy-related complications. Laparoscopic common bile duct exploration (LCBDE) is a valuable and cost-effective method for treating gallstones and choledocholithiasis (CCL) using a single-stage approach.^[5,6]^ Primary closure is preferred to avoid T-tube-related complications. A history of gastrectomy poses a significant challenge because of extensive adhesions and the altered gastrointestinal anatomy, which may increase the risk of organ injury and technical difficulty. Therefore, surgical common bile duct (CBD) exploration can be performed as the last choice.

The Hem-o-lok clip is used during laparoscopic surgery as a substitute ligation material and it is considered inert, nonconductive, and compatible with computed tomography (CT) and magnetic resonance imaging. Since its introduction, it has been widely used and is currently considered safe. However, we noticed that the use of Hem-o-lok clips has led to various complications. Although these complications are infrequent, some may be fatal and should be taken into consideration. One potential complication is the migration of the clip into the CBD, resulting in stone formation. Herein, we report a case of Hem-o-lok clip migration into the CBD after multiple upper abdominal surgeries. The reporting of this study conforms to the CARE guidelines.^[7]^

## 2 Case presentation

**Figure 1:**
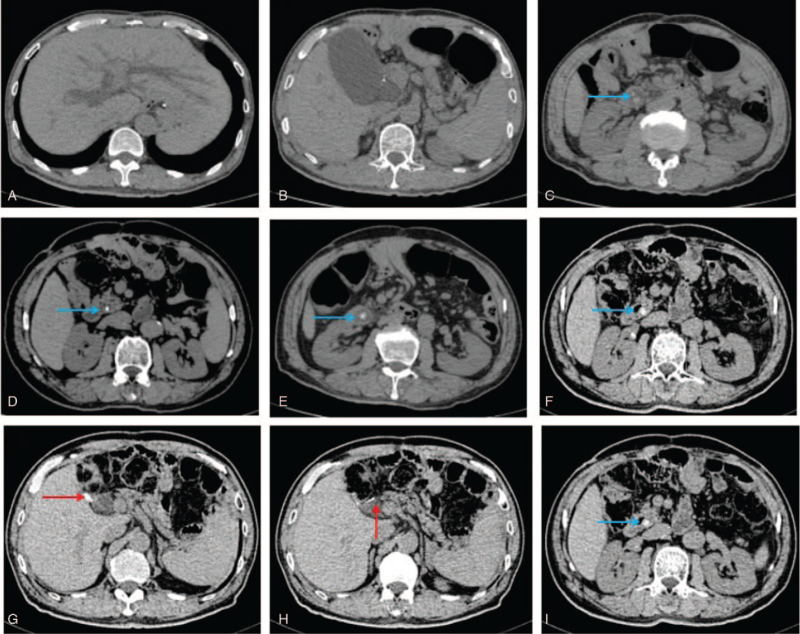
Computed tomography findings. (A) Computed tomography before the first laparoscopic common bile duct exploration (LCBDE) showing a dilated intrahepatic bile duct. (B) Enlarged gallbladder with a thickened wall. (C) Dilated common bile duct (1.5 cm in width) with stones in the distal aspect (blue arrow). Extensive adhesions were present under the incision. (D) A calcified shadow (1.0 cm in length) was present 6 months after LCBDE without symptoms. (E) A circular low-density shadow around the calcified shadow 9 months after the previous operation. (F) The calcified shadow enlarged to a diameter of 1.0 cm 12 months after the LCBDE. (G, H) Three clips placed (red arrow): 2 clips were placed on the cystic duct and 1 clip was placed on the cystic artery; dilation of the common bile duct is noted (1.5 cm in width). (I) A calcified shadow (1.0 cm in length) in the distal common bile duct (blue arrow).

A 67-year-old man was admitted to our hospital with a 12-hour history of acute right upper abdominal pain. The patient had undergone Billroth II gastrectomy in June 2012 to treat stage IIIB gastric adenocarcinoma (T4aN2M0) with 6 courses of adjuvant chemotherapy. Close follow-up, including gastric endoscopy, revealed cardia cancer in March 2017. The patient then underwent total gastrectomy. Emergency percutaneous transhepatic gallbladder drainage had been performed for acute obstructive suppurative cholangitis 17 months prior to this admission (Fig. 1 A–C), and LCBDE with T-tube had been performed 5 months later. The T-tube was removed 3 months after LCBDE with no residual stones in CBD confirmed by T-tube cholangiography. The follow-up CT revealed CBD stones 6 months after LCDBE (Fig. 1 D–F). The patient received no surgical treatment due to asymptomatic and low platelets after chemotherapy. He reported no history of nicotine or alcohol abuse. He also had no history of hypertension, diabetes, or coronary heart disease. Physical examination revealed abdominal tenderness, a tinge of jaundice, and fever (maximum temperature, 38.3°C). The abnormal laboratory data were as follows: white blood cell count, 2.94 × 1012/L (reference range, 4.0–9.0 ×1012/L); neutrophilic granulocyte percentage, 0.79 (0.5–0.7); gamma-glutamyl transferase, 437.5 U/L (10–60 U/L); total bilirubin, 79 μmol/L (3.4–17.1 μmol/L); direct bilirubin, 52 μmol/L (0.0–6.8 μmol/L); aspartate aminotransferase, 264.2 U/L (15–40 U/L); and alanine aminotransferase, 393.4 U/L (9–50 U/L). The CT examination also revealed a dilated CBD (1.5 cm) and a 1.0-cm-long high-density shadow at the end of the CBD (Fig. 1 G–I). There were extensive abdominal adhesions at the original gastrectomy incision site; they were evaluated by ultrasonography. The lateral movement of the intestines was <1 cm, and the longitudinal movement was <3 cm under normal respiration. Considering that the patient had no history of incisional hyperplasia, intestinal obstruction, or abdominal infection, the extent of the adhesions was assigned a score of 3.^[8,9]^ The duodenal papilla could not be identified using gastrointestinal endoscopy.

**Figure 2:**
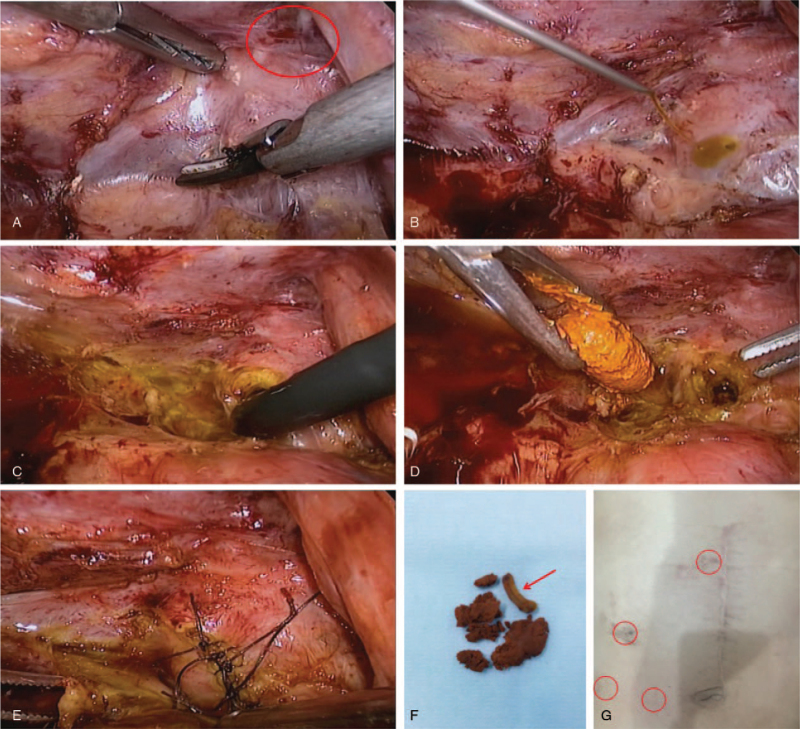
Surgical procedure. (A) The adhesions were dissected to expose the upper edge of the duodenum as a marker to find the first hepatic hilum (Hem-o-lok clips are indicated by a red circle). (B) Fine-needle aspiration was performed to identify the common bile duct. (C) Choledochoscope insertion. (D) Extraction of the stone. (E) Primary closure of the common bile duct. (F) While splitting the stone, a Hem-o-lok clip was found. (G) Trocar distribution.

**Figure 3:**
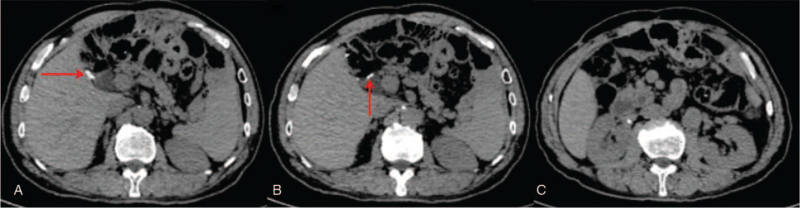
Postoperative computed tomography findings. (A, B) Computed tomography showed that the three clips (red arrow) had not moved. (C) Gradual dilation of the common bile duct (1.2 cm in width) without stone formation at the 12-month follow-up.

**Figure 4:**
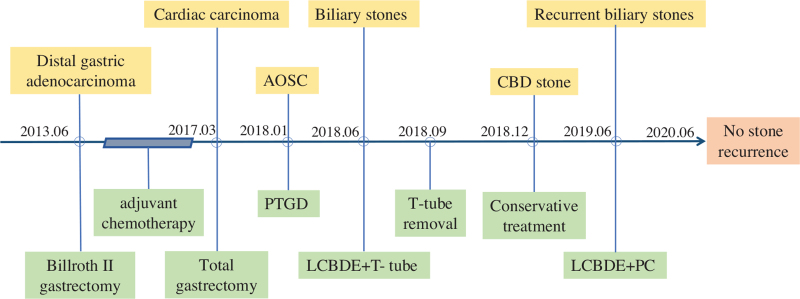
Timeline of the patient's history, surgeries, and follow-up. AOSC = acute obstructive suppurative cholangitis, LCBDE + PC = laparoscopic common bile duct exploration with primary closure, LCBDE + T-tube = laparoscopic common bile duct exploration with T-tube placement, PTGD = percutaneous transhepatic gallbladder drainage.

The patient underwent a repeat LCBDE. A carbon dioxide pneumoperitoneum of 12 mm Hg was created via a 10-mm port inserted on the right side of the umbilicus (3 cm from the incision). One additional trocar (12.5 mm) was positioned in the para-xiphoid region, and 2 5-mm ports were placed in the right hypochondrium. The adhesion was then carefully dissected from the right to the midline after separating the duodenum from the liver, and the CBD and clips were exposed. Fine-needle aspiration confirmed the CBD. The stone had formed around a Hem-o-lok clip, which served as the core of the stone extracted by choledochoscopy. The normal ampullary sphincter was assured with no stone residue, and the CBD incision was intermittently sutured with 5–0 polydioxanone (Fig. 2 A–G). The duration of the operation was 117 min. The patient recovered well and had no postoperative complications, such as bleeding or bile leakage, and he was discharged on postoperative day 6. Twelve months later, CT showed that all the 3 clips were still in their original locations, with no stone recurrence in the CBD (Fig. 3 A–C). The timeline of the history, surgeries, and follow-up was shown in Figure 4.

## 3 Discussion

The incidence of gallstones is higher after gastrectomy than in the general population. Approximately 10% of patients with gallstones have concomitant CCL^[10]^ which is associated with severe complications, such as acute pancreatitis and cholangitis. A complex interaction between the disconnected nerve supply and decreased cholecystokinin secretion is considered an essential factor.

Concomitant cholecystectomy during gastrectomy is unnecessary^[11]^ due to the low rate of severe gallstone-related complications. The administration of ursodeoxycholic acid can significantly reduce the incidence of gallstones after gastrectomy.^[12]^ However, gallstone management decisions after gastrectomy are mainly dependent on the surgeon's preference and expertise. A history of gastrectomy has long been considered a significant challenge for endoscopists; procedures in such cases must be performed by highly experienced and skilled endoscopists.^[13]^ Meanwhile, ERCP is not a benign procedure, and complications, such as pancreatitis, duodenal perforation, and bleeding, occur with a higher frequency in these patients^[14]^. With advancements in laparoscopic techniques, LCBDE has proven feasible and practical. Kim et al^[15]^ noted that LCBDE + LC should be the initial approach for patients with CCL and a history of gastrectomy. Additionally, LCBDE maintains both the structural and functional integrity of the sphincter of Oddi, helping to avoid bile juice regurgitation and reduce the recurrence of gallstones or the occurrence of cholangitis. It is worth noting that the conventional LCBDE using a T-tube is associated with various complications, including peritoneal biliary infection, water and electrolyte metabolism disturbances, T-tube displacement, and inconvenience related to prolonged T-tube placement.^[16]^ Upon completion of LC, primary closure of the CBD is safe, feasible, and provides better short- and long-term outcomes.^[17–19]^

Previous gastrectomy is a relative contraindication for LCBDE because of extensive adhesions. Preoperative evaluation of adhesions is required to achieve successful insertion of the initial trocar. We followed the history-taking process with ultrasound assessment and added precise CT evaluation to visualize the adhesions. The open insertion method is highly recommended for the first trocar. Careful anatomical assessment and dissection techniques are essential to avoid organ damage. Using the right-side approach to expose the duodenum and separate it from the hepatic hilum, the CBD can be exposed and identified by needle aspiration of bile. Choledochoscopy enables complete clearance of stones and ensures the absence of residual stones. Primary closure should be performed for a dilated CBD (diameter >0.8 cm) in cases without severe infections.

Recurrence of CCL is usually associated with cholestasis, abnormal biliary dynamics, and CBD infection.^[20]^ In the present case, no residue stone in the CBD was found during the T tube cholangiography 3 months after LCBDE, however, a 6-month follow-up CT showed a CBD stone. The stone was found to form around a Hem-o-lok clip during the bile duct re-exploration. Therefore, we hypothesized that the Hem-o-lok clip may have been the factor that induced stone recurrence. Several different foreign bodies, including clips and sutures, have been implicated in providing a nidus for stone formation in the biliary tree. Hem-o-lok clip migrated into the CDB with subsequent stone formation.

Postoperative clip migration is a rare but well-established complication of LC. The clip may migrate at any time, but the median time is 2 years after LC.^[21]^ In our case, however, the time of clip migration was three to 6 months. Most patients present with typical symptoms of primary CCLs. However, the exact pathophysiology remains unknown. Many factors, such as inadequate clip placement, a high number of clips, bile leakage, sterile inflammation, and local necrosis, contribute to clip migration. We did not find a T-tube fistula during the re-exploration of the CBD. After removing the T-tube, slight bile leakage might have occurred, inducing migration of the clip into the CBD. Moreover, we found that the clip was clinging to the CBD and provoked a chronic rejection response mechanism, leading to fistula formation around the clip and extending into the CBD. The routine use of nonabsorbable clips is safe and effective, and alternatives can further reduce the risk. The clips should be accurately placed and should not be too close to the CBD. Some surgeons advocate harmonic scalpel dissection of the cystic artery and duct instead of using clips.^[22,23]^ The use of absorbable clips is another option.^[24]^ One absorbable clip can replicate the outcomes obtained using metal clips.^[25]^ Many authors recommend the use of absorbable sutures^[26,27]^ or silk sutures^[28]^ for cystic duct ligation.

Stones associated with clip migration after LC are often extracted using ERCP, with a high success rate.^[21]^ In most cases, only a single ERCP attempt is required for successful clearance. Biliary strictures and huge stones are essential factors that may contribute to the failure of ERCP. However, when ERCP fails or in patients with a history of gastrectomy, LCBDE can serve as an alternative for safe and efficient stone extraction. Open surgery is the final treatment choice.

## 4 Conclusion

Although LCBDE with primary closure can be performed successfully for certain patients with CCL who have a history of gastrectomy and LCBDE, careful case evaluation and selection are necessary. The Hem-o-lok clip should be placed appropriately to ensure that it is not too close to the CBD. Absorbable clips or sutures can be used as alternatives for ligation.

## Acknowledgments

We would like to thank Editage (www.editage.com) for English language editing.

## Author contributions

CJ contributed to the data analysis and interpretation and drafting of the manuscript. MW, SL, and GW contributed to the data collection and analysis. XL and GW contributed to the manuscript's data interpretation and critical revision for important intellectual content. All authors have read and approved the manuscript.

**Conceptualization:** Chao Jiang.

**Data curation:** Xueyan Liu, Shuxuan Li.

**Formal analysis:** Guangzhen Wu.

**Supervision:** Guangyi Wang, Meng Wang.

**Writing – original draft:** Chao Jiang.

**Writing – review & editing:** Guangyi Wang, Meng Wang.
